# Quality of Life Assessment of Post-Cholecystectomy Patients in Iquitos, Perú

**DOI:** 10.51894/001c.164024

**Published:** 2026-07-31

**Authors:** Olivia Dunmore, Derek Esty, Sophie Bair, Lauren Gillette, Brad Draher, Grace Larsen, Cydney Pittenger, Kisa Seerveld, Travis Gordon

**Affiliations:** 1 College of Osteopathic Medicine, Michigan State University

## 17

### Introduction

Cholecystectomy is one of the most common abdominal surgeries in the world and can result in a subset of various gastrointestinal symptoms known as post-cholecystectomy syndrome (PCS) in 5-47% of cases. Additionally, cholecystectomy can increase the risk of depression and general reduction in quality of life (QoL), though this component is less understood.

### Objectives

Our study aims to examine adult patients seeking medical attention at the “Clinica San Martin de Porres” in Iquitos, Peru, who have undergone cholecystectomy. We sought to understand the general quality of life and mental health of patients who have had a cholecystectomy.

### Methods

After informed consent was obtained, 25 patients in Iquitos, Peru, with a history of cholecystectomy answered three questionnaires read aloud in Spanish: The Gastrointestinal Quality of Life Index (GIQLI), the Patient Health Questionnaire-9 (PHQ-9), and the Generalized Anxiety Disorder-7 (GAD-7).

### Results

The mean GIQLI for all participants was 118.8, the mean PHQ-9 for all participants was 9.8, and the mean GAD-7 for all participants was 9.1. Six of the 25 patients (24%) were found to have post-cholecystectomy diarrhea (PCD). This subpopulation was found to have significantly less favorable quality of life outcomes, including GIQLI of 108.5 vs. 122.2 in those without PCD, PHQ-9 score of 11.3 vs. 9.3 in those without PCD, and GAD-7 score of 11.2 vs. 8.4 in those without PCD.

### Discussion

Analysis of our small post-cholecystectomy patient population suggests a mildly decreased QoL, evidenced by a GIQLI score of 118.8, mild depression by PHQ-9, and mild anxiety by GAD-7. Patients found to have PCD had a significantly lower QoL at 108.5. They also were shown to have moderate levels of depression and anxiety by PHQ-9 and GAD-7, respectively, and were the subpopulation with the lowest overall mental health parameters. Further assessment with larger prospective trials will be necessary to determine if these trends are valid in larger populations.

